# Commentary: Debating secularism: a liberal cosmopolitan perspective

**DOI:** 10.3389/fsoc.2023.1193232

**Published:** 2023-07-05

**Authors:** Tariq Modood

**Affiliations:** Centre for the Study of Ethnicity and Citizenship, SPAIS, University of Bristol, Bristol, United Kingdom

**Keywords:** multiculturalism, secularisms, moderate secularism, radical secularism, ethnoreligious groups

I thank Haldun Gülalp for discussing my views on secularism and multiculturalism (Gülalp, 2023, p. 2). He is not in agreement with them but that may be because he is not always correct in his understanding of them. There is a strong binary at work in how he presents my thinking, which I believe is not present in my work and which Gülalp does not show is present.

*Pace* Gülalp I don't hold a primordialist view of ethnocultural groups (p. 2) I argue they are being continually (re-)created and so I often speak of post-immigration ethnoreligious formations, which are dialectically shaped from, one might say, the outside in and from the inside out (Modood, 2019). Nor do I think that “rights and recognition must be granted not to individuals but to communities” (p. 2); not just to individuals but also to communities would be more accurate. He attributes to me the view that religious organizations should be “taking over some of the tasks of the state”, whilst I say that they may be supported to supplement state provision in education, health care and so on if they have something to offer, such as a faith dimension, which is wanted by some citizens and which the state cannot supply.

Whilst Gülalp defends what he calls “classical secularism”, what I call radical or statist secularism, I defend moderate secularism, one of the most common forms of secularism across Europe today, though more in some countries than others (Sealy and Modood, 2022). While religious organizations should respect that the state has its own raison d'etre and should not be subordinated to religion, in moderate secularism the state does not confine religion to a restricted private space if religion can contribute to the public good, just as the state supports economic organizations, public broadcasting, cultural and sporting institutions, science, universities and so on, while respecting (at least in liberal democracies) their relative autonomy (Modood, 2019).

These are significant differences between us, partly based on misinterpretations of my views and partly by my supporting a secularism that is inclusive of religion in the public sphere rather than simply a guarantor of freedom of conscience.

Even more fundamentally, I cannot accept Gülalp's understanding of multiculturalism. His suggestion that Young's (1990) and Kymlicka's (1995) championing the inclusion of groups such as “Jews, blacks, Asians, Indians, Mexicans” is based on “[t]he only thing in common between these distinct social groups is that they all experience some form of discrimination… [and] have nothing to do with cultures” (p. 3) is quite mistaken. Kymlicka believes that membership in a culture I can identify as my own is essential to individual autonomy. Young goes even further and argues that asserting one's group identity including overthrowing identities that others—including secularists—want to impose on you is a fundamental process of liberation and equality. While my own multiculturalism is not the same as theirs I too insist that equal citizenship consists in allowing all groups, especially the oppressed or marginalized, to promote their positive identity, which other citizens and the state have a duty to recognize through appropriate institutional accommodation and differential treatment, if necessary. This in fact is the multiculturalist contribution to the idea of equal citizenship. Unlike Young and the early Kymlicka, I have extended this idea to include religious identity groups such as, say, British Muslims, and thus argued that multiculturalism is incompatible with radical secularism; it is however compatible with moderate secularism, suitably multiculturalized.^1^

Gülalp goes on to note that some religious groups may be closed mind and even anti-democratic. Yes, but not all religious groups are so one cannot dismiss the possibility of any religious groups being a respected feature of the public space. Moreover, many radical secularists—Soviet and Chinese communists, for instance—exhibit the same characteristics that Gülalp attributes here to religious adherents.

While I have argued that religious groups like Muslims in the West can be racialized in the same way that Jews, another religious minority, are racialized, Gülalp argues that religion and race are quite distinct because religious identity is not ascribed in modern societies, where people may negotiate their own religious identities and children of mixed marriages are free to choose their own identity (p. 3). In fact, racial identities are not as fixed as Gülalp believes. The way black and Asian identities have been asserted, promoted, negotiated, transmuted in the US and the UK in the last few decades makes that evident; and of course children are born within racially mixed marriages not just religiously mixed marriages and in neither case are they free to choose their own identity without having to negotiate or resist the prevalent (changing) social meanings ascribed to identities like theirs and their parents.

“[F]ixing religion (or sexual orientation) as the primary identity of a group of citizens would weaken and impoverish their participation in civic life” (p. 4). Yet nowhere have I suggested that, say a Hindu, should always have their religious identity as their primary one (or a lesbian should have that identity as her primary one). I note however there are contexts, social and political, and in institutions like schools and universities, where some people themselves say that about their identities. Without encouraging monistic and separatist identities, I want to allow people to be able to assert what they believe to be their primary identity and how institutions, policies and laws have to adapt around them. Above all, I believe it is contrary to equal citizenship to say that people may assertively seek civic recognition for their gender, sexual and racial but not religious identities. Such exceptionalizing of religion is one of the principal differences between moderate and radical secularisms.

Having brought out our differences, let me conclude with a measure of agreement. Gülalp says: “The answer to the question of policy should not start from the community defined top-down by the state, but from the citizens endowed with rights, who can create their own communities from the bottom-up.” I very much agree with this and it is one of the reasons that I prefer the UK's moderate secularism that is in principle willing to speak to an independent, bottoms-up Muslim Council of Britain rather than the French radical secularists top-down approach that creates and disbands its own organizations, such as Conseil Français du Culte Musulman, for dialogue with Muslims.

## Author contributions

The author confirms being the sole contributor of this work and has approved it for publication.

## Footnotes

^1^More recently, Kymlicka has changed his mind and come to the view that “all of the arguments for adopting multiculturalism as a way of tackling the legacies of ethnic and racial hierarchies apply to religion as well” (Kymlicka, 2015, p. 28).

## References

[B1] Gülalp H. (2023). Debating secularism: a liberal cosmopolitan perspective. Front. Sociol. 8, 113208. 10.3389/fsoc.2023.1113208 36938138 PMC10017520

[B2] Kymlicka W. (1995). Multicultural Citizenship: A Liberal Theory of Minority Rights. Oxford: Clarendon Press.

[B3] Kymlicka W. (2015). “The three lives of multiculturalism” in Revisiting Multiculturalism in Canada: Theories, Policies, Debates, eds Guo S. Wong L. (Sense Publishers), 17–35.

[B4] Modood T. (2019). Essays on Secularism and Multiculturalism. London: ECPR and Rowman and Littlefield International.

[B5] Sealy T. Modood T. (2022). Diversities and dynamics in the governance of religion: inter-regional comparative themes. Religion State Soc. 50, 469-485. 10.1080/09637494.2022.2132079

[B6] Young I. M. (1990). Justice and the Politics of Difference. Princeton, NJ: Princeton University Press.

